# Associations Between Lifestyle Behaviors and Handgrip Strength in Community-Dwelling Older Women: A Cross-Sectional Study

**DOI:** 10.3390/healthcare14152269

**Published:** 2026-07-24

**Authors:** Yuji Maruyama, Maho Ueda

**Affiliations:** Faculty of Sport and Health Science, Tokai Gakuen University, Miyoshi 470-0207, Japan; ueda-ma@tokaigakuen-u.ac.jp

**Keywords:** handgrip strength, older women, lifestyle factors, social interaction, physical function, sarcopenia

## Abstract

**Highlights:**

**What are the main findings?**

- Meal enjoyment, social interaction, and outing frequency were associated with handgrip strength after adjustment for age.
- Higher lifestyle scores were associated with higher handgrip strength.

**What are the implications of the main findings?**

- Information on meal enjoyment and social engagement may provide useful contextual information regarding lower handgrip strength among older women.
- These cross-sectional findings support considering multiple lifestyle behaviors together but do not establish causal relationships.

**Abstract:**

**Background/Objectives**: Handgrip strength is a widely used indicator of physical function that is associated with various health outcomes of older adults. However, the relationship between lifestyle factors and handgrip strength, as well as the age-associated relationship between them, remains insufficiently understood. This study examined age-adjusted associations between multiple lifestyle factors and handgrip strength among older women. **Methods**: During this cross-sectional study of 2206 older women, handgrip strength was categorized into low, middle, and high tertiles. Lifestyle factors such as meal enjoyment, exercise frequency, sleep quality, social interaction, and outing frequency were assessed using a questionnaire. Group differences were evaluated using an analysis of variance and chi-square tests. A general linear model with age as a covariate was used to compare age-adjusted proportions of lifestyle behaviors across handgrip strength tertiles. **Results**: Participants in the high handgrip strength tertile were younger and more likely to report favorable lifestyle behaviors. After adjusting for age, meal enjoyment (*p* = 0.024), social interaction (*p* = 0.001), and outing frequency (*p* = 0.017) remained significantly associated with handgrip strength. In contrast, sleep quality (*p* = 0.073) and exercise frequency (*p* = 0.060) were not significantly associated with handgrip strength after age adjustment. Higher lifestyle scores were significantly associated with higher handgrip strength. **Conclusions**: Among older women, meal enjoyment, social interaction, and outing frequency were associated with handgrip strength after adjustment for age. These findings highlight the potential relevance of meal enjoyment and social engagement as contextual lifestyle characteristics associated with handgrip strength. However, causal relationships cannot be inferred because of the cross-sectional study design.

## 1. Introduction

Handgrip strength is widely recognized as a simple and reliable indicator of overall muscle strength and physical function in older adults [1,2,3]. It has been extensively used as a clinical and epidemiological marker associated with adverse health outcomes, including frailty, disability, hospitalization, and mortality [4,5]. Large-scale population-based studies have also demonstrated strong associations between grip strength and all-cause mortality as well as chronic diseases [6].

Aging is accompanied by a progressive decline in muscle strength that is more pronounced in women than in men. Older women are particularly vulnerable to declines in muscle strength due to factors such as low baseline muscle mass, hormonal changes, and lifestyle-related factors. Therefore, identifying lifestyle factors associated with handgrip strength in older women is important for identifying modifiable factors associated with physical function.

Lifestyle factors play an important role in maintaining physical function in older adults. Previous studies have shown that physical activity, dietary habits, and sleep quality are associated with muscle strength and physical performance [7,8,9]. Additionally, social interaction and participation in daily activities contribute to physical function and well-being [10,11,12,13]. Multiple lifestyle behaviors also jointly influence physical function [14,15].

However, most previous studies have examined individual lifestyle behaviors in isolation, and few have simultaneously examined multiple lifestyle domains [16,17,18]. Moreover, the relationships between lifestyle behaviors and handgrip strength may be confounded by age, which is strongly associated with both lifestyle behaviors and muscle strength. Therefore, it is important to examine these associations while appropriately accounting for age. Furthermore, evidence specifically focused on community-dwelling older women is limited. Individuals who actively participate in community-based activities may exhibit distinct lifestyle characteristics that influence their physical function. Therefore, this study aimed to examine the associations between multiple lifestyle behaviors, including meal enjoyment, exercise frequency, sleep quality, social interaction, and outing frequency, and handgrip strength among community-dwelling older women. We also examined these associations after adjusting for age. We hypothesized that healthier lifestyle behaviors would be associated with higher handgrip strength.

## 2. Materials and Methods

### 2.1. Study Design and Participants

**Figure 1:**
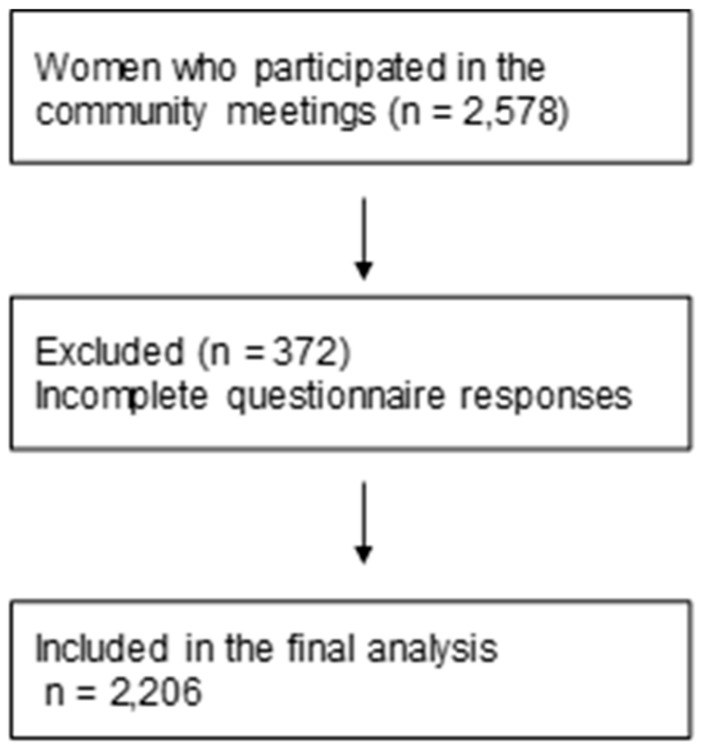
Flow diagram of participant recruitment and selection.

This cross-sectional study included community-dwelling older women who participated in community-based meetings coordinated by the Matsuyama City Social Welfare Council between May and December 2024. Eligibility criteria for participation in the meetings included being 60 years of age or older and not being certified as requiring long-term care under the Japanese Long-Term Care Insurance System. A total of 2578 women participated in the meetings. After excluding 372 participants with incomplete questionnaire responses, 2206 participants were included in the present study. The participant recruitment and selection process is shown in Figure 1. Because participants with missing data were excluded only from the corresponding analyses, the number of participants varied slightly across analyses depending on data availability.

### 2.2. Ethical Considerations

The original data collection for this study was conducted under Ethics Approval No. 2022-19, approved by the Ethics Committee of Tokai Gakuen University on 30 December 2022, prior to participant recruitment and data collection.

Subsequently, Ethics Approval No. 2025-21 was approved by the Ethics Committee of Tokai Gakuen University on 17 December 2025 to permit secondary analyses and publication of the existing dataset.

All study procedures were conducted in accordance with the principles of the Declaration of Helsinki. Written informed consent was obtained from all participants prior to participation.

### 2.3. Assessment of Handgrip Strength

Handgrip strength was measured using a digital handgrip dynamometer (T.K.K. 5401; Takei Scientific Instruments Co., Ltd., Niigata, Japan). Measurements were conducted by trained staff following a standardized measurement protocol based on the Japanese Ministry of Education, Culture, Sports, Science and Technology physical fitness test. To reduce the physical burden and potential risk to community-dwelling older adults during community-based health assessments, grip strength was measured once for each hand rather than twice for each hand, as recommended in the original protocol. The average of the left- and right-hand measurements was used for the analyses. Measurements were performed with participants standing and their arms naturally at their sides. Participants were instructed to exert maximal effort during each measurement.

### 2.4. Classification of Handgrip Strength

Participants were categorized into tertiles (low, middle, and high) based on the distribution of handgrip strength among women, using the 33.3rd and 66.7th percentiles (cut-off values: 18.65 kg and 22.05 kg).

### 2.5. Assessment of Lifestyle Factors

Lifestyle factors were assessed using a structured self-administered questionnaire with predefined response categories. The questionnaire assessed exercise frequency, sleep quality, meal enjoyment, social interaction, and outing frequency. Exercise frequency was assessed using the question, “How many times per week do you exercise?” with four response options (≥5 days/week, 3–4 days/week, 1–2 days/week, and <1 day/week). Participants reporting exercise ≥3 days/week were categorized as having a high exercise frequency, whereas those reporting exercise <3 days/week were categorized as having a low exercise frequency. Sleep quality was assessed using the question, “How would you rate your sleep quality?” with four response options (good, fairly good, fairly poor, and poor). Responses of good or fairly good were categorized as good, whereas fairly poor or poor were categorized as poor. Meal enjoyment was assessed using the question, “How would you rate the enjoyment of your meals?” with four response options (very enjoyable, fairly enjoyable, not very enjoyable, and not enjoyable). Responses of very enjoyable or fairly enjoyable were categorized as high meal enjoyment, whereas responses of not very enjoyable or not enjoyable were categorized as low meal enjoyment. Social interaction was assessed using the question, “How often do you have opportunities to talk with other people, including family members?” with four response options (many, somewhat many, somewhat few, and few). Responses of many or somewhat many were categorized as high, whereas those of somewhat few or few were categorized as low. Outing frequency was assessed using the question, “How many times per week do you go outside?” with four response options (≥5 days/week, 3–4 days/week, 1–2 days/week, and <1 day/week). Participants reporting outings ≥3 days/week were categorized as active, whereas those reporting outings <3 days/week were categorized as inactive.

### 2.6. Lifestyle Score

A composite lifestyle score was calculated by summing the number of favorable lifestyle factors (exercise, sleep, meal enjoyment, social interaction, and outing frequency). Each favorable lifestyle factor was assigned one point because the lifestyle score was intended to provide a simple composite indicator of favorable lifestyle behaviors. The total score ranged from 0 to 5, with higher scores indicating more favorable lifestyle behaviors. The lifestyle score was categorized into three groups according to the distribution of the scores: low (0–2 points), middle (3 points), and high (4–5 points).

### 2.7. Statistical Analysis

Descriptive statistics were calculated for all variables. Continuous variables were presented as mean ± standard deviation (SD), and categorical variables were presented as numbers and percentages.

Differences among handgrip strength tertiles were assessed using one-way analysis of variance (ANOVA) for continuous variables and the chi-square test for categorical variables.

To compare age-adjusted proportions of favorable lifestyle behaviors across handgrip strength tertiles, the General Linear Model procedure was applied with age as a covariate. Binary lifestyle variables were coded as 0 or 1, and age-adjusted proportions and 95% confidence intervals were estimated from the estimated marginal means. These analyses were conducted as age-adjusted group comparisons and were not intended as predictive models of handgrip strength. The association between lifestyle score categories and handgrip strength tertiles was evaluated using the chi-square test. A linear trend across lifestyle score categories was assessed using the linear-by-linear association test.

Participants with missing data for each variable were excluded from the corresponding analyses.

All statistical analyses were performed using IBM SPSS Statistics version 30.0 (IBM Corp., Armonk, NY, USA). A two-tailed *p* < 0.05 was considered statistically significant.

## 3. Results

Characteristics of the participants in each handgrip strength tertile are presented in Table 1.

For age, post hoc comparisons were performed using the Bonferroni correction; all pairwise comparisons were significant (*p* < 0.001).

Mean age differed significantly across tertiles. Participants in the low handgrip strength tertile were older than those in the middle and high handgrip strength tertiles (82.4 ± 6.0 vs. 78.6 ± 5.8 vs. 75.5 ± 6.0 years, respectively).

Exercise frequency did not differ significantly across tertiles (*p* = 0.065). In contrast, sleep quality differed significantly across tertiles. The proportion of participants who reported good sleep quality in the high tertile was higher than that in the low tertile (82.3% vs. 76.7%; *p* = 0.021). Meal enjoyment was also significantly associated with handgrip strength. The proportion of participants who reported high meal enjoyment in the middle and high tertiles was higher than that in the low tertile (97.1% and 97.0% vs. 93.8%; *p* = 0.001).

Social interaction also differed significantly across tertiles. The proportion of participants who reported high levels of social interaction increased from the low tertile to the high tertile (low, 58.2%; middle, 66.9%; high, 72.8%; *p* = 0.001). Outing frequency was also significantly associated with handgrip strength (*p* = 0.001).

**Figure 2:**
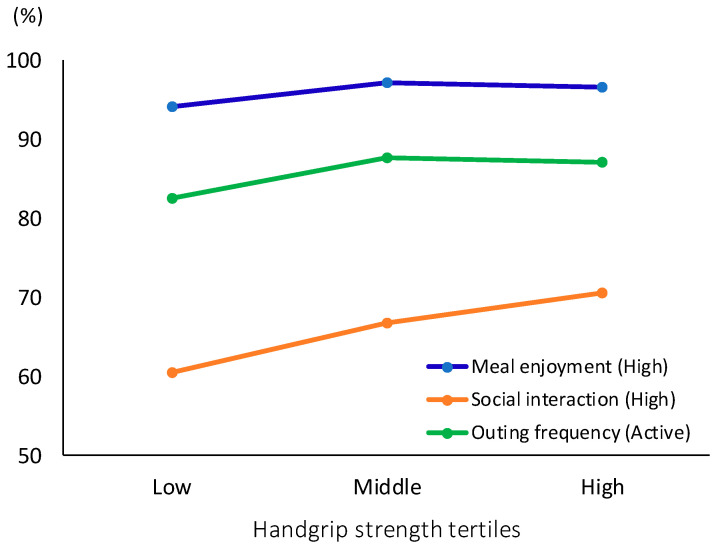
Age-adjusted proportions of significantly associated lifestyle factors according to handgrip strength tertiles.

After adjusting for age (Table 2), meal enjoyment (*p* = 0.024), social interaction (*p* = 0.001), and outing frequency (*p* = 0.017) remained significantly associated with handgrip strength; however, sleep quality (*p* = 0.073) and exercise frequency (*p* = 0.060) did not. These age-adjusted associations are presented in Figure 2.

**Figure 3:**
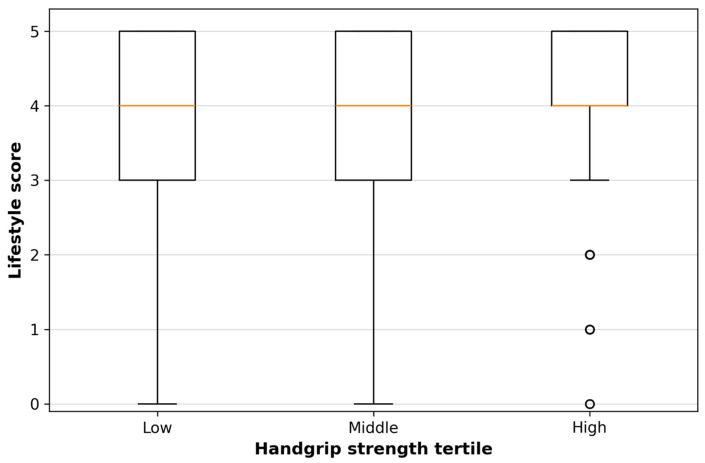
Distribution of lifestyle scores according to handgrip strength tertiles. The box plots show the median and interquartile range (IQR). Whiskers extend to 1.5 × IQR, and circles indicate outliers.

Figure 3 shows the distribution of lifestyle scores across tertiles.

**Figure 4:**
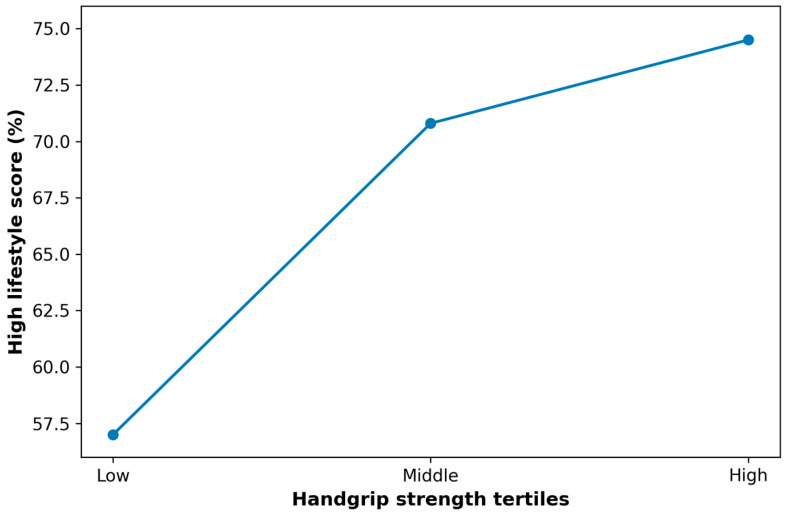
Proportion of participants with high lifestyle scores according to handgrip strength tertiles.

Additionally, lifestyle score categories (low: 0–2 points; middle: 3 points; and high: 4–5 points) were significantly associated with handgrip strength (Table 3). Participants with higher lifestyle score categories were more likely to belong to the higher handgrip strength tertiles (*p* < 0.001). The proportion of participants with high lifestyle scores according to handgrip strength tertiles is shown in Figure 4.

## 4. Discussion

This study examined the associations between multiple lifestyle factors and handgrip strength among community-dwelling older women. The main finding was that meal enjoyment, social interaction, and outing frequency were significantly associated with handgrip strength after adjustment for age.

Meal enjoyment was significantly associated with handgrip strength after adjustment for age. Previous studies have reported associations between nutritional factors and muscle function in older adults [19,20,21,22,23,24]. However, the questionnaire item used in the present study assessed the subjective enjoyment of meals and did not directly measure dietary intake, diet quality, or nutritional adequacy. Therefore, the present finding should be interpreted as an association between meal enjoyment and handgrip strength rather than as evidence of an association between objective nutritional status and handgrip strength.

Similarly, social interaction and outing frequency were significantly associated with handgrip strength after adjustment for age. These findings are consistent with previous studies indicating that social engagement is associated with better health outcomes [25,26,27]. Reduced outing frequency has also been linked to functional decline in older adults [10,28,29].

Participants with higher lifestyle scores tended to have higher handgrip strength. However, because of the cross-sectional design, causality cannot be inferred. This observation is consistent with previous studies reporting that multiple healthy lifestyle behaviors have combined and cumulative effects on health outcomes [30,31]. In contrast, sleep quality and exercise frequency were not significantly associated with handgrip strength after adjusting for age. The attenuation of the association between sleep quality and handgrip strength after age adjustment suggests that this relationship may be largely explained by age. Age-related changes in sleep patterns, including reduced sleep efficiency and increased sleep fragmentation, are common among older adults and may partly explain this finding. Therefore, the apparent association between sleep quality and handgrip strength observed before age adjustment may have reflected age-related changes in both sleep quality and physical function. Similarly, the lack of a significant association between exercise frequency and handgrip strength may reflect limitations of the exercise assessment. The questionnaire assessed only exercise frequency and did not capture important characteristics such as exercise intensity, duration, or type. In particular, resistance training is more strongly associated with muscle strength than exercise frequency alone. Furthermore, participants with the same exercise frequency may differ substantially in the intensity, duration, and type of exercise performed. Therefore, the absence of a significant association in the present study should not be interpreted as evidence that exercise is unrelated to muscle strength. These findings highlight that multiple lifestyle behaviors were associated with handgrip strength among community-dwelling older women. The composite lifestyle score used in this study assigned equal weight to each lifestyle behavior to provide a simple and practical summary of overall lifestyle characteristics rather than to estimate the relative physiological importance of individual behaviors. Because the primary aim of the score was to capture the accumulation of healthy lifestyle behaviors, all components were treated equally. Although individual lifestyle behaviors may differ in their relative contributions to physical function, the present score was intended as an exploratory indicator of multidimensional healthy lifestyle behaviors rather than a weighted index of their physiological importance. Future studies should examine alternative scoring approaches, including weighted scores based on the relative contributions of individual lifestyle factors.

This study had several strengths. First, it included a relatively large sample of community-dwelling older women, enabling stable statistical analysis. Second, multiple lifestyle domains were assessed simultaneously, thus providing a comprehensive evaluation of factors associated with physical function. Third, age-adjusted analyses were conducted because age is a major confounding factor.

However, several limitations of this study should be acknowledged. First, because of the cross-sectional design, causal relationships could not be established; therefore, the findings should be interpreted with caution. Second, participants were recruited from community-based meetings coordinated by a Social Welfare Council and may have represented a relatively health-conscious and socially active population compared with the general population of older women. Because comparable national demographic data were not available, the extent of this potential selection bias could not be quantitatively assessed. Accordingly, caution is warranted when generalizing the findings to older women with frailty, severe functional limitations, or social isolation. Third, this study was based on a secondary analysis of an existing dataset. Therefore, information on other potentially important social determinants, such as socioeconomic status, educational background, living arrangements, and social support, was not available. These unmeasured factors may also be associated with handgrip strength and should be considered in future studies. Fourth, grip strength was measured once for each hand using a standardized protocol to minimize participant burden during community-based health assessments. However, formal assessment of device calibration and intra-rater reliability was not conducted, which represents a methodological limitation. In addition, because this study was a secondary analysis of an existing dataset including all eligible participants, an a priori sample size calculation was not performed. This should be considered when interpreting the study findings. Fifth, lifestyle factors were assessed using a self-reported questionnaire; therefore, recall bias could not be ruled out.

Despite these limitations, meal enjoyment, social interaction, and outing frequency among older women were associated with handgrip strength after adjustment for age. These findings indicate that multidimensional lifestyle factors, particularly meal enjoyment and social engagement, are associated with handgrip strength among community-dwelling older women. However, these findings should be interpreted and generalized with caution because of the characteristics of the study population. Future longitudinal studies are needed to confirm these associations.

## 5. Conclusions

In conclusion, meal enjoyment, social interaction, and outing frequency were associated with handgrip strength after adjustment for age. Higher lifestyle scores were also associated with higher handgrip strength. Information on multiple lifestyle behaviors may provide useful contextual information regarding characteristics associated with lower handgrip strength among older women. However, these findings should not be interpreted as evidence of a validated screening tool or causal relationships. Future longitudinal studies are needed to confirm these associations.

## Figures and Tables

**Table 1:** Participant characteristics according to handgrip strength tertiles in older women.

Variable	Total (n = 2206)	Low (n = 732)	Middle (n = 725)	High (n = 749)	*p*-Value
Age, years	78.8 ± 6.6	82.4 ± 6.0	78.6 ± 5.8	75.5 ± 6.0	0.001
Exercise frequency, n (%)					
High	1329 (61.5)	413 (58.3)	459 (64.2)	457 (62.1)	0.065
Low	831 (38.5)	296 (41.7)	256 (35.8)	279 (37.9)	
Sleep quality, n (%)					
Good	1719 (79.0)	552 (76.7)	561 (78.0)	606 (82.3)	0.021
Poor	456 (21.0)	168 (23.3)	158 (22.0)	130 (17.7)	
Meal enjoyment, n (%)					
High	2088 (96.0)	675 (93.8)	699 (97.1)	714 (97.0)	0.001
Low	88 (4.0)	45 (6.3)	21 (2.9)	22 (3.0)	
Social interaction, n (%)					
High	1431 (66.0)	420 (58.2)	476 (66.9)	535 (72.8)	0.001
Low	737 (34.0)	302 (41.8)	235 (33.1)	200 (27.2)	
Outing frequency, n (%)					
Active	1854 (85.8)	562 (79.6)	630 (87.9)	662 (89.8)	0.001
Inactive	306 (14.2)	144 (20.4)	87 (12.1)	75 (10.2)	

Values are presented as mean ± standard deviation (SD) or number (percentage). *p*-values were calculated using one-way ANOVA for continuous variables and the chi-square test for categorical variables. Due to missing data, the number of participants varies across variables.

**Table 2:** Age-adjusted proportions of favorable lifestyle factors according to handgrip strength tertiles.

Variables	Low	Middle	High	*p*-Value	n
Meal enjoyment (high)	94.2 (92.7–95.8)	97.1 (95.6–98.5)	96.6 (95.1–98.1)	0.024	2176
Sleep quality (good)	77.0 (73.9–80.2)	78.0 (75.0–81.0)	82.0 (78.9–85.1)	0.073	2175
Exercise frequency (high)	57.9 (54.1–61.7)	64.2 (60.6–67.8)	62.4 (58.7–66.1)	0.060	2160
Social interaction (high)	60.6 (57.0–64.2)	66.8 (63.3–70.2)	70.6 (67.0–74.1)	0.001	2168
Outing frequency (active)	82.6 (79.9–85.3)	87.7 (85.2–90.2)	87.1 (84.5–89.7)	0.017	2160

Values are presented as age-adjusted proportions and 95% confidence intervals estimated using the General Linear Model with age as a covariate. Sample sizes varied slightly across lifestyle factors because participants with missing data were excluded only from the corresponding analyses.

**Table 3:** Association between lifestyle score and handgrip strength tertiles.

Lifestyle Score	Low (n = 732)	Middle (n = 725)	High (n = 748)	*p*-Value
Low	136 (18.6%)	88 (12.1%)	73 (9.8%)	
Middle	179 (24.5%)	124 (17.1%)	118 (15.8%)	
High	417 (57.0%)	513 (70.8%)	557 (74.5%)	<0.001

Lifestyle score categories were defined as follows: low, 0–2 points; middle, 3 points; and high, 4–5 points. *p*-values were calculated using the chi-square test. A significant linear trend was also observed (*p* < 0.001).

## Data Availability

The data presented in this study are available on request from the corresponding author. However, the data are not publicly available due to ethical and privacy restrictions, as they contain information related to human participants. Data sharing is subject to approval by the relevant institutional ethics committee and compliance with institutional regulations.

## References

[1] Marín-Jiménez N., Bizzozero-Peroni B., Molina-García P., Ortega F.B., Chaput J.-P., Zhang K., Lang J.J., McGrath R., Tomkinson G.R., Martínez-Vizcaíno V. (2025). Clinical importance of simple muscular fitness tests to predict long-term health conditions: A systematic review and meta-analysis of 94 cohort studies. Br. J. Sports Med..

[2] Tomkinson G.R., Lang J.J., Rubín L., McGrath R., Gower B., Boyle T., Klug M.G., Mayhew A.J., Blake H.T., Ortega F.B. (2024). International norms for adult handgrip strength: A systematic review of data on 2.4 million adults aged 20 to 100+ years from 69 countries and regions. J. Sport Health Sci..

[3] McGrath R., Vincent B.M., Hackney K.J., Robinson-Lane S.G., Downer B., Clark B.C. (2020). The Longitudinal Associations of Handgrip Strength and Cognitive Function in Aging Americans. J. Am. Med. Dir. Assoc..

[4] López-Bueno R., Andersen L.L., Koyanagi A., Núñez-Cortés R., Calatayud J., Casaña J., Del Pozo Cruz B. (2022). Thresholds of handgrip strength for all-cause, cancer, and cardiovascular mortality: A systematic review with dose-response meta-analysis. Ageing Res. Rev..

[5] Polo-López A., Andersen L.L., Núñez-Cortés R., López-Bueno R., Cruz-Montecinos C., Suso-Martí L., Calatayud J. (2026). Handgrip strength asymmetry increases risk of all-cause and cardiovascular mortality: A dose-response analysis across 28 countries. Prog. Cardiovasc. Dis..

[6] Celis-Morales C.A., Welsh P., Lyall D.M., Steell L., Petermann F., Anderson J., Iliodromiti S., Sillars A., Graham N., Mackay D.F. (2018). Associations of grip strength with cardiovascular, respiratory, and cancer outcomes and all cause mortality: Prospective cohort study of half a million UK Biobank participants. BMJ.

[7] Baik I. (2025). Associations of dietary and physical activity behaviors with handgrip strength in middle-aged and older adults: A prospective cohort study. Sci. Rep..

[8] Hasan F., Tu Y.-K., Lin C.-M., Chuang L.-P., Jeng C., Yuliana L.T., Chen T.-J., Chiu H.-Y. (2022). Comparative efficacy of exercise regimens on sleep quality in older adults: A systematic review and network meta-analysis. Sleep Med. Rev..

[9] Li Y., Quan W., Gao Z., Wang X., Wang Y., Meng H., Kang J. (2025). Effects of exercise interventions on subjective sleep quality in older adults: A systematic review and meta-analysis of studies using the Pittsburgh Sleep Quality Index. Front. Med..

[10] Nakou A., Dragioti E., Bastas N.-S., Zagorianakou N., Kakaidi V., Tsartsalis D., Mantzoukas S., Tatsis F., Veronese N., Solmi M. (2025). Loneliness, social isolation, and living alone: A comprehensive systematic review, meta-analysis, and meta-regression of mortality risks in older adults. Aging Clin. Exp. Res..

[11] Zhang H., Hao X., Qin Y., Yang Y., Zhao X., Wu S., Li K. (2025). Social participation classification and activities in association with health outcomes among older adults: Results from a scoping review. J. Adv. Nurs..

[12] Monteiro J.M., Gonçalves R., Bastos A., Barbosa M.R. (2023). Social engagement and wellbeing in late life: A systematic review. Ageing Soc..

[13] Zhou J., Yang Y., Li S., Mao N., Chen X., Wang D., Zhang Y., Shi X., Li J., Gao X. (2025). Patterns of social participation among older adults and their association with self-rated health: The mediating role of activities of daily living. Aging Clin. Exp. Res..

[14] Seino S., Nofuji Y., Yokoyama Y., Abe T., Nishi M., Yamashita M., Narita M., Hata T., Shinkai S., Kitamura A. (2023). Combined impacts of physical activity, dietary variety, and social interaction on incident functional disability in older Japanese adults. J. Epidemiol..

[15] Qiao L., Wang Y., Deng Y., Peng J., Li Y., Li M., Tang Z. (2025). Combined healthy lifestyle behaviors and all-cause mortality risk in middle-aged and older US adults: A longitudinal cohort study. Arch. Gerontol. Geriatr..

[16] Koemel N.A., Biswas R.K., Ahmadi M.N., Teixeira-Pinto A., Hamer M., Rezende L.F.M., Mitchell J., Leech R.M., Sawan M., Allman-Farinelli M. (2026). Minimum combined sleep, physical activity, and nutrition variations associated with lifespan and healthspan improvements: A population cohort study. eClinicalMedicine.

[17] Nam H.-K., Jang S.-N., Kawachi I., Ma Y., Cho S.-I. (2025). Association between physical activity and handgrip strength among older adults in the Korean longitudinal study of ageing. Sci. Rep..

[18] Ma X., Zhao Y., Zhao H., Zhou D., Ai H., Sun M. (2026). Association between the planetary health diet and sleep health in older adults: Findings from a national community-based study. Front. Nutr..

[19] Yang Y., Pan N., Luo J., Liu Y., Ossowski Z. (2025). Exercise and nutrition for sarcopenia: A systematic review and meta-analysis with subgroup analysis by population characteristics. Nutrients.

[20] Granic A., Sayer A.A., Cooper R., Robinson S.M. (2025). Nutrition in the prevention and treatment of skeletal muscle ageing and sarcopenia: A single nutrient, a whole food and a whole diet approach. Proc. Nutr. Soc..

[21] Li M.-L., Zhang F., Luo H.-Y., Quan Z.-W., Wang Y.-F., Huang L.-T., Wang J.-H. (2024). Improving sarcopenia in older adults: A systematic review and meta-analysis of randomized controlled trials of whey protein supplementation with or without resistance training. J. Nutr. Health Aging.

[22] Heo S., Yang S.J. (2025). Dietary factors and nutritional guidelines for sarcopenia in older adults: A narrative review. Korean J. Community Nutr..

[23] Moslemi E., Mansouri F., Khudayqulov B., Mahmoodi M., Alavi S.M., Mahmoudi-Zadeh M., Souni F., Askarpour M., Nouri M., Shateri Z. (2025). The relationship between elderly dietary index and sarcopenia: A case-control study. BMC Geriatr..

[24] Ruiz-Valenzuela R.E., Artacho R., Ruiz-López M.D., Molina-Montes E. (2025). Healthy dietary patterns and risk of sarcopenia in adults aged >50 years: A systematic review and meta-analysis considering EWGSOP1 and EWGSOP2 criteria. Nutrients.

[25] World Health Organization (2025). From Loneliness to Social Connection: Charting a Path to Healthier Societies.

[26] Abugroun A., Shah S.J., Covinsky K., Hubbard C., Newman J.C., Fang M.C. (2025). Low social engagement and risk of death in older adults. J. Am. Geriatr. Soc..

[27] Luo L., Xing Y., Shang Z., Ren W., Zhang L. (2025). Association between diversified social interaction and health among older adults in China: A longitudinal analysis by interaction type and frequency. BMC Geriatr..

[28] Kanamori S., Kai Y., Aida J., Kondo K., Kawachi I., Hirai H., Shirai K., Ishikawa Y., Suzuki K. (2014). Social Participation and the Prevention of Functional Disability in Older Japanese: The JAGES Cohort Study. PLoS ONE.

[29] Holt-Lunstad J., Smith T.B., Baker M., Harris T., Stephenson D. (2015). Loneliness and Social Isolation as Risk Factors for Mortality: A Meta-Analytic Review. Perspect. Psychol. Sci..

[30] Ding D., Van Buskirk J., Nguyen B., Stamatakis E., Elbarbary M., Veronese N., Clare P.J., Lee I.-M., Ekelund U., Fontana L. (2022). Physical Activity, Diet Quality and All-Cause, Cardiovascular Disease and Cancer Mortality: A Prospective Study of 346,627 UK Biobank Participants. Br. J. Sports Med..

[31] Nyberg S.T., Singh-Manoux A., Pentti J., Madsen I.E.H., Sabia S., Alfredsson L., Bjorner J.B., Borritz M., Burr H., Goldberg M. (2020). Association of Healthy Lifestyle With Years Lived Without Major Chronic Diseases. JAMA Intern. Med..

